# Radiological evolution of autograft fat used for skull base reconstruction after transsphenoidal surgery for pituitary adenomas

**DOI:** 10.1007/s11102-022-01210-6

**Published:** 2022-02-22

**Authors:** Giulia Cossu, Victoire Turin-Huet, Marta Garvayo Navarro, Georgios Papadakis, Roy Thomas Daniel, Vincent Dunet, Mahmoud Messerer

**Affiliations:** 1 Department of Neurosurgery, Lausanne University Hospital and University of Lausanne, Lausanne, Switzerland; 2 Faculty of Biology and Medicine, University of Lausanne, Lausanne, Switzerland; 3 Department of Endocrinology, Lausanne University Hospital and University of Lausanne, Lausanne, Switzerland; 4 Department of Medical Radiology, Lausanne University Hospital and University of Lausanne, Lausanne, Switzerland

**Keywords:** CSF leakage, Fat autograft, Pituitary adenomas, Skull base reconstruction, Transsphenoidal surgery

## Abstract

**Purpose:**

Cerebro-spinal fluid leak after transsphenoidal surgery for pituitary adenomas may be prevented by skull base reconstruction with fat autograft. However, graft changes may interfere with the interpretation of postoperative images. Our aim is to describe the radiological evolution of the fat autograft.

**Methods:**

A retrospective analysis was performed, including patients undergoing a transsphenoidal surgery for pituitary adenomas with a fat autograft for skull base reconstruction. Clinical and radiological data were collected, with assessment of fat autograft and extent of resection. Statistical analysis was performed using Kruskal–Wallis and Wilcoxon signed-rank test while Spearman’s Rho was used to analyze the relationship between variables.

**Results:**

Seventy-two patients were included. Macroadenomas were diagnosed in 62 cases (86.1%) and in 21 cases an invasion of the cavernous sinus was described (29%). Gross total resection was achieved in 84.7% of cases. The volume of the fat graft significantly decreased between 3 months and 1 year after surgery (p = 0.01) and between 1 year and the last follow-up (mean 4.63 years, p < 0.01). Fat signal ratio significantly diminished between 3 months and 1 year in unenhanced and enhanced T1-weighted sequences (p = 0.04 and p = 0.02 respectively). Volume reduction was related to the decrease in signal ratio in unenhanced T1 sequences (p = 0.008).

**Conclusion:**

Fat resorbs with time: almost 50% of the fat volume is lost during the first year after surgery and 60% is resorbed at 4.6 years. T1-signal, before and after gadolinium injection, also decreases during the first year, probably because of the progressive fibrosis of the graft. This information will contribute to the interpretation of postoperative images.

## Introduction

Sellar tumors are frequent and represent 10–15% of intracranial neoplasms. Pituitary adenomas (PA) are the most frequent pituitary tumors, accounting for more than 90% of cases [1]. The management of the vast majority of PA, except prolactinomas, is surgical, with the aim of obtaining a gross total resection (GTR) with decompression of visual structures and normal pituitary and biological remission with functioning tumors [1–3]. The transsphenoidal approach represents the gold standard for the surgical treatment of these tumors, as it proved to be associated with excellent outcomes in terms of tumor control, visual and endocrinological outcomes [3–8]. Postoperative morbidity and mortality rates are low: major morbidity rates are between 1 and 2% and CSF leak is found in 2–9% of cases [9–12]. When a peroperative CSF leak is detected, careful reconstruction is mandatory in order to avoid a postoperative CSF leak with potential further complications like meningitis. One of the most common methods for reconstruction is the use of autologous fat graft, usually harvested from the patient’s abdomen [13, 14]. The fat graft is positioned in the sella, reinforced with fibrin glue to seal the arachnoid hole. Autologous fat grafting has proved to be a reliable technique to avoid a postoperative CSF leak, but its presence may interfere with the interpretation of postoperative images to evaluate the presence of residual tumor. One of the main issues is the unclear resorption rate of the fat tissue and its contrast enhancement, making the differentiation between scar tissue, residual tumor or packing material extremely challenging.

The aim of this study is to evaluate and describe the radiological evolution of the sellar fat autograft after endonasal transsphenoidal surgery in order to provide a reference for the interpretation of postoperative pituitary images and therefore allow a differentiation between residual tumor versus grafting material. To our knowledge, this is the first study in literature that systematically analyzes the radiological evolution of autograft fat used for reconstruction after transsphenoidal pituitary surgery.

## Materials and methods

We performed a retrospective analysis of our consecutive surgical series of patients undergoing a transsphenoidal approach for PA at the Neurosurgical Department of Lausanne University Hospital between January 2007 and December 2018. We included only the patients who underwent fat autografting for skull base reconstruction during surgery. Exclusion criteria were: patients who underwent previous surgeries in other institutions, patients with no skull base reconstruction or with reconstructions performed with other materials, patients undergoing transcranial approaches and finally, patients with less than 1 year of postoperative follow-up. An ethical approval was obtained before starting the study.

Medical records were retrospectively reviewed in order to extract the demographic and clinical data, surgical data, radiological data and follow-up data to detail the postoperative course and eventual complications. Radiological follow-up is standardized in our institution with pituitary MRI performed at 3 months, 1 year and 2 years postoperatively. Examinations were performed on different 1.5 or 3 T MRI scanners (all Siemens, Erlangen, Germany) over the study period. Our pituitary imaging protocol included 1.5-mm thick slices (or 2-mm on 1.5 T scanners) with unenhanced sagittal T1-weighted spin-echo, coronal T2-weighted, dynamic coronal T1-weighted spin echo, and enhanced sagittal and coronal T1-weighted spin-echo sequences after injection of gadolinated contrast media. Radiological assessment of the fat autograft and completeness of resection was performed by an experienced neuroradiologist. For each PA the cranio-caudal long axis in cm and volume in mm^3^ as well as the invasion of the cavernous sinus according to the Knosp classification were recorded. Gross total resection (GTR) was defined as macroscopically complete resection, with no residual tumor visible at the 3 months postoperative MRI. For each patient the fat autograft was manually delineated on each time-point to obtain its volume in mm^3^. Autograft signal was evaluated on all sequences by placing a 5-mm^2^ ellipsoid region-of-interest (ROI) on the center of the graft, and a similar ROI on the pons to calculate the signal ratio (i.e. signal of the graft ROI divided by the signal of the pons ROI). Signal ratio was preferred to absolute value to account for inter-scanners and inter-patients’ variability. All data was extracted and assembled in a coded database. Statistical analysis of the data was performed with Stata/IC 16.1 software (StataCorp, Texas, USA). Continuous variables are presented as mean ± standard deviation (SD), and categorical variables as number and percentage. Fat autograft volume and signal ratios were compared between the time-points using Kruskal–Wallis test and Wilcoxon signed-rank test, while their relationship was evaluated through Spearman’s Rho. The significance level was set at a p value < 0.05.

## Results

**Fig. 1:**
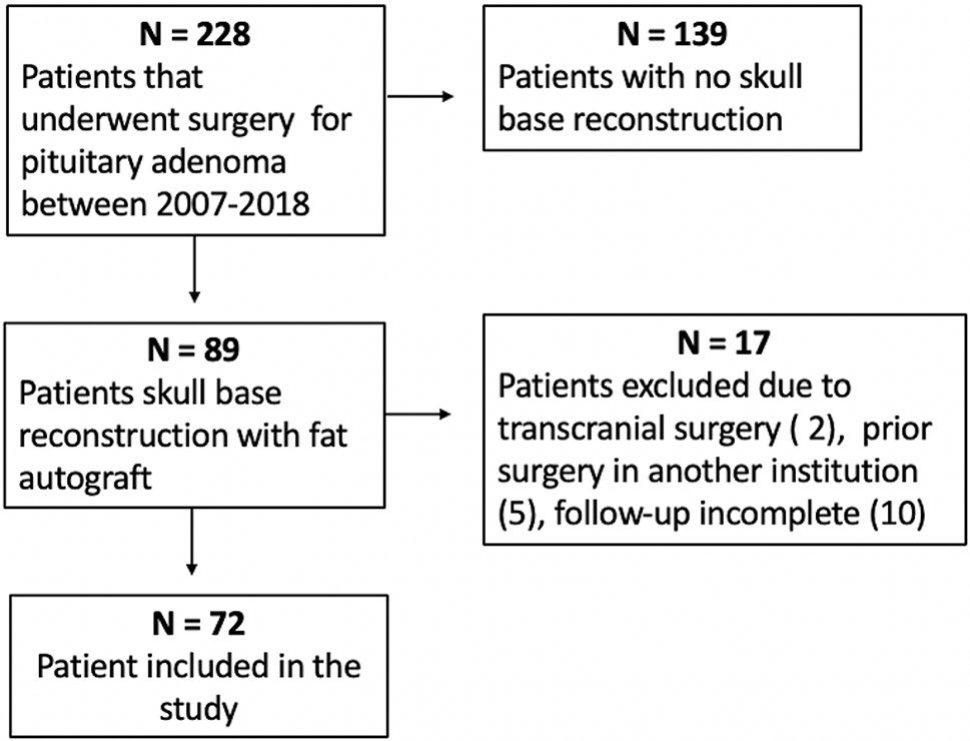
Flow chart showing the selection process for patients’ inclusion

In the aforementioned period, 228 patients underwent endoscopic endonasal transsphenoidal surgery for PA. Of those, 89 patients (39%) had a skull base reconstruction with fat autograft. Seventeen patients were excluded because of previous transsphenoidal surgery in other institutions, performance of transcranial approaches and follow-up shorter than 1 year. Finally, 72 patients were eligible for our study (Fig. 1). Our population included 32 men (44%) and mean age at the time of surgery was 52.7 ± 16.7 years (range:14–85 years). Forty-seven patients (65.3%) had non-functioning tumors and 25 (34.7%) had functioning PA. Within the functioning tumors, 12 patients had a GH-secreting (48%) and 8 had ACTH-secreting PitNETs (32%) resulting in Cushing’s disease. Three patients presented a prolactinoma (12%) and 2 patients a TSH-secreting PitNET (8%). In most of cases, a macroadenoma was diagnosed (62 cases, 86.1%), while in 10 cases a microadenoma was detected (13.9%). Mean cranio-caudal diameter was 2.13 ± 1.17 cm and mean volume 7803 ± 8351 mm^3^. Regarding the invasion of the cavernous sinus, 12.5% adenomas were Knosp 0 (9 cases), 20.8% Knosp 1 (15 cases), 37.5% Knosp 2 (27 cases), 18.1% Knosp 3 (13 cases) and 11.1% Knosp 4 (8 cases). Clinical and radiological characteristics of the population included are summarized in Table 1.

**Table 1:** The demographic and radiological characteristics of the population included are here detailed, as well as the surgical outcome. The number of patients is indicated in brackets

Mean age	52.7 years	± 16.7 SD
Clinical presentation	Headaches	19.44% (14)
	Visual disturbances	44.44% (32)
	Secretory syndromes	31.94% (23)
	Hormonal deficiency	34.72% (25)
	Apoplexy	12.5% (9)
	Incidental finding	5.55% (4)
Adenoma size	Macroadenoma	86.11% (62)
	Microadenoma	13.8% (10)
Hormonal secretion	Non-functioning PA	65.27% (47)
	Functioning PA	34.72% (25)
Knosp grade	Knosp 0	12.5% (9)
	Knosp I	20.8% (15)
	Knosp II	37.5% (27)
	Knosp III	18.1% (13)
	Knosp IV	11.1% (8)
GTR	Yes	84.7% (61)
	No	15.3% (11)

*PA pituitary adenoma*

As defined by our inclusion criteria, a transsphenoidal approach was performed for PA with skull base reconstruction with fat autograft in all patients. Routine packing of the sella with a fat autograft, even without a perioperative CSF leak was performed in our institution between 2007 and 2012. After that period, fat was used as a packing material only in cases of observed or presumed arachnoid rupturing during surgery. GTR was achieved in 84.7% of cases (61 patients), as confirmed by the postoperative MRI at 3 months after surgery (Table 1).

**Fig. 2:**
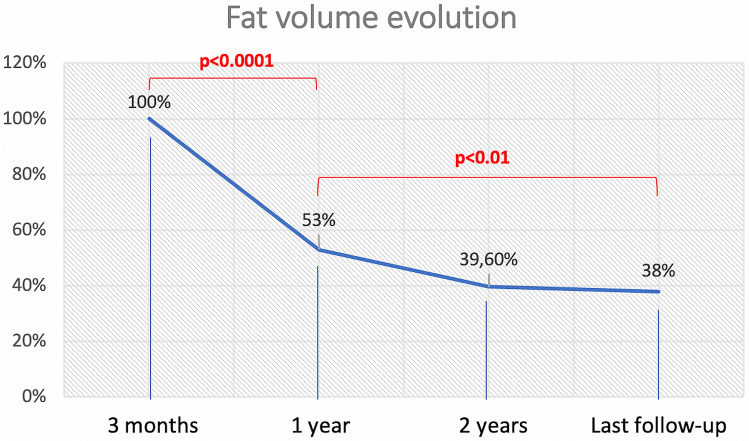
Graphic of the fat volume evolution. The % represents the volume remaining at 3 months, 1 year, 2 years and at last follow-up (mean 4.6 years). We can appreciate that the most significant change occurs during the first year after surgery, with the loss of almost 50% of the initial volume (p < 0.0001). The fat resorption continues in a slower rate after this period and the difference becomes significant when compared to last follow-up (p < 0.01)

Eight patients out of 228 (3.5%) presented with postoperative CSF leaked necessitating a second surgery for sellar packing. A 46% decrease of the fat graft volume was observed when comparing the MRI performed at 3 months and at 1 year after surgery. Fat resorption continued at a slower rate after one year, with loss of 59.4% of the volume at 2 years. Residual fat volume at last follow-up (mean 4.64 ± 2.23 years) was decreased by 60.2%, as shown in the graphic (Fig. 2). A statistically significant difference was evident between the MRI performed at 3 months and at 1 year (p < 0.0001) and between the images performed at 1 year and at last follow-up (p < 0.01). Thus, the significant period of fat decrease is within the first year after surgery and the process continues then slowly during the long-term follow-up.

**Fig. 3:**
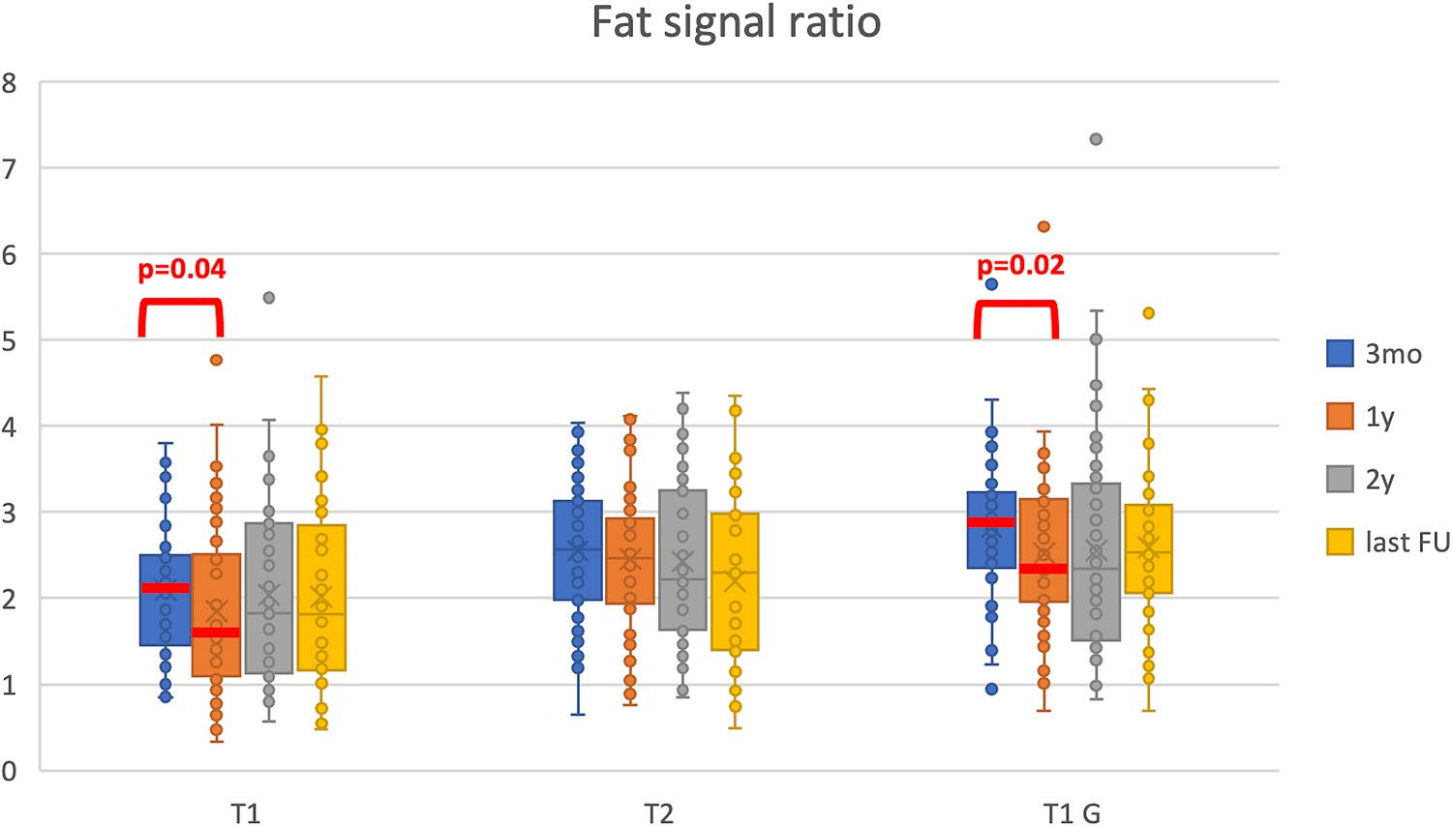
Sequential analysis of the signal ratio of the fat autograft are here detailed. Signal ratios on unenhanced T1-weighted images and enhanced T1-weighted images after contrast media injection decreased significantly between 3-months and 1-year MRI (p = 0.037 and p = 0.022, respectively). They remained stable over time thereafter. The signal was relatively stable on T2-weighted images. *3mo* MRI at 3 months, *1y* MRI at 1 year, *2y* MRI at 2 years, *Last FU* MRI at last follow-up (mean 4.6 years), *T1* T1-weighted MRI (unenhanced), *T1 G* T1-weighted MRI with gadolinium administration, *T2* T2-weighted MRI

Sequential analyses of the signal ratio of the fat autograft did not show any significant change on T2-weighted images. Instead, signal ratios on unenhanced T1-weighted images and enhanced T1-weighted images after contrast media injection decreased significantly between 3-months and 1-year MRI (p = 0.037 and p = 0.022, respectively). They remained stable over time thereafter (Fig. 3).

The volume loss between 3 months and 1 year was positively correlated with decrease of signal ratios on T1-weighted images (Spearman’s Rho = 0.23, p = 0.008), while no correlation was reported with signal ratios in enhanced T1-weighted images after gadolinium administration.

## Discussion

Transsphenoidal surgery has proven to be a safe and effective approach of PA, with a mortality rate < 1% and of severe complications < 2% [12, 15]. Nevertheless, complications still occur, and postoperative CSF leakage due to the intraoperative rupture of the arachnoid during tumor resection remains one the most common, reported in between 2 and 9% of surgeries [9, 10, 12].

Given the need to reconstruct and pack the sella and sphenoid sinus in order to prevent a postoperative CSF rhinorrhea, autologous adipose tissue grafting has been used for the last decades in neurosurgery [14, 15]. Fat autografting has proved to be a reliable, safe and effective technique, with a complication rate of 1% [13, 15, 16]. However, its use has also brought up new challenges concerning the radiological interpretation of the postoperative images in terms of evaluation of residual tumor.

Precise literature data on the resorption process of the fat autograft is missing and sparse studies report the fat autograft in the sella may still be present 10 years after surgery [17]. Our study shows that the most significant decrease in fat volume occurs during the first year after surgery. Indeed, almost half (46.2%) of the fat is resorbed between the MRI performed at 3 and 12 months postoperatively. This process continues then at a slower rate during the long-term follow-up, with more than 60% of the fat resorbed at a mean of 4.6 years. Detailed knowledge of the radiological evolution of the fat graft and being aware that the critical resorption period takes place during the first year after the grafting, will facilitate the interpretation of the postoperative images of the pituitary region.

Therefore, some studies have shown that fat grafts present post-gadolinium enhancement in T1-weighted images [18–20], which complicates the radiological interpretation and can easily be mistaken for residual tumor. The physiology behind the fat graft contrast enhancement has been studied and it is probably due to an adaptation of the fat to the recipient environment with enhanced vascularization for the survival of the fat graft [21, 22]. The fat contrast enhancement was also confirmed in our study, with a significant decrease of signal ratio between 3-months and 1-year MRI on unenhanced T1-weighted images and enhanced T1 -weighted images after gadolinium injection (Fig. 3). This decrease in signal ratios on T1-weighted MRI was related to the reduction in fat graft volume over time between 3 months and 1 year and this might be due to the progressive fibro-inflammatory involution of the graft with a more evident hypointensity of the graft and also a decrease in contrast enhancement (not significant). This phenomenon should be kept in mind as the fibrosis could be misinterpreted as recurrent tumor. A careful analysis of the preoperative images is fundamental to localize the initial tumor and then to identify the autograft fat in early postoperative images. A strong collaboration between the neurosurgeon, the neuroradiologist and the endocrinologist is mandatory for an optimal interpretation of the postoperative images. The results of this study could help in the interpretation of the postoperative images after transsphenoidal surgery for pituitary adenomas when fat graft is used for skull base reconstruction. To our knowledge, this is the first study describing the evolution of free fat grafts in the sella after pituitary surgery. Nevertheless, the study has its limitations, mainly due to the reduced number of patients and to the limited number of cases with residual tumor, which prevented an analysis of the relative signal of the residual tumor. Furthermore, we did not routinely perform an early postoperative MRI in this surgical series. Thus, fat signal and volume at baseline were not analyzed. Larger studies with long-term follow-up are needed in order to validate our results.

## Conclusions

Fat grafting for skull base reconstruction after pituitary surgery is a useful technique in the prevention of CSF leak but it may interfere with the interpretation of the postoperative imaging, making challenging the differentiation between the packing material and residual or recurrent tumor, as scarce data exist on the radiological evolution of the harvested fat. Our results concerning the resorption rate of the fat graft showed that almost 50% of the volume is lost during the first year after surgery and subsequently a steady-state is reached with 60% of the initial volume being resorbed at last follow-up at 4.6 years. The signal on MRI also decreases on unenhanced and enhanced T1-weighted sequences between 3 months and 1 year and this reduction in signal ratio in unenhanced sequences was associated with reduction of the autograft volume. This valuable information will contribute to guide the interpretation of the sellar contents after surgery. Larger studies are required to confirm our findings.

## Abbreviatons

3mo: MRI at 3 months
1y: MRI at 1 year
2y: MRI at 2 years
Last FU: MRI at last follow-up (mean 4.6 years)
T1: T1-weighted MRI (unenhanced)
T1 G: T1-weighted MRI with gadolinium administration
T2: T2-weighted MRI
